# Presence of new mutations in the *TP53* gene in patients with low-risk myelodysplastic syndrome: two case reports

**DOI:** 10.1186/s13256-017-1301-8

**Published:** 2017-05-21

**Authors:** Fernando Barroso Duarte, Romélia Pinheiro Gonçalves Lemes, Talyta Ellen de Jesus dos Santos, Maritza Cavalcante Barbosa, João Paulo Leitão de Vasconcelos, Francisco Dário Rocha-Filho, Ilana Zalcberg, Diego Coutinho, Monalisa Feliciano Figueiredo, Luciana Barros Carlos, Paulo Roberto Leitão de Vasconcelos

**Affiliations:** 1 Department of Surgery, Federal University of Ceará, Fortaleza, Ceara Brazil; 2 Research Laboratory in Hemoglobinopathies and Genetics of Hematologic Diseases, Federal University of Ceará, Capitão Francisco Pedro street, n. 1210, Rodolfo Teófilo, Fortaleza, Ceara Brazil; 3 Laboratory of Molecular Biology-Center for Bone Marrow Transplantation, CEMO-National Cancer Institute - INCA, Rio de Janeiro-Rio de Janeiro, Brazil; 4 Center of Hematology and Hemotherapy of Ceara – Cryobiology Laboratory, Fortaleza, Ceara Brazil

**Keywords:** Myelodysplastic syndromes, *TP53* mutations, Prognosis

## Abstract

**Background:**

Myelodysplastic syndromes are heterogeneous disorders. Patients with myelodysplastic syndrome disease often have ineffective hematopoiesis, cytopenias, blood cell dysplasia in one or more cell types, and are at high risk for developing acute myeloid leukemia. In myelodysplastic syndrome, mutations of *TP53* gene are usually associated with complex karyotype and confer a worse prognosis. In the present study, two mutations in this gene are presented and discussed with the clinical evolution of the patients.

**Case presentation:**

The first case is a 77-year-old Brazilian woman diagnosed as having multiple lineage dysplasia myelodysplastic syndrome according to World Health Organization 2016 and classified as very low-risk by Revised International Prognostic Scoring. The second case is an 80-year-old Brazilian man also diagnosed as having multiple lineage dysplasia myelodysplastic syndrome and classified as low risk. The mutation described in the first case was already identified in some neoplasias and it is associated with a poor prognosis, but it had never been reported before in myelodysplastic syndrome. The second mutation has never been described.

**Conclusions:**

This is a novel report for the scientific community and may be very helpful as we can better understand the disease and the impact of mutations through the follow-up of these patients and others in the future. Both patients are in a good clinical condition, suggesting that these mutations may not alter the clinical course of the disease or may be associated with a good prognosis, but their role in the disease must be investigated more deeply in a larger population.

## Background

Myelodysplastic syndromes (MDS) are heterogeneous disorders. The *TP53* gene is implicated in the manifestation of hematologic features, specifically macrocytic anemia, frequently involved in MDS. Mutations in the *TP53* gene are usually associated with complex karyotype and confer a worse prognosis. Somatic mutations in the *TP53* tumor suppressor gene are found in approximately 50% of all human tumors, making it the most commonly mutated gene. The expression of p53 protein and the study of mutations are important tools, mainly for the prognosis of MDS [1].

**Table 1:** Clinical and hematological features of both patients

	Mutation	
	c.394A > T	c.783-1_784delGTG
Age (years)	79	82
Gender	Female	Male
Complete blood count		
Hemoglobin, g/dL	10.3	10.82
Hematocrit, %	30.0	33.0
Mean corpuscular volume (fL)	88.08	92.5
Leukocytes (×10^9^/L)	5.5	4.59
Neutrophils(×10^9^/L)	3.3	0.77
Platelets (×10^9^/L)	208	113.2
Karyotype	46,XX	46,XY
Bone marrow blasts	0.5%	2.2%
Bone marrow fibrosis	Degree II	Without fibrosis
Bone marrow iron	Normal	Normal
Bone marrow cellularity	Hypocellular	Hypercellular
CD34 immunoreactivity	Negative	Negative
p53 immunohistochemistry	Negative	Positive (2%)
Transfusion dependence	No	No

Although the biological and clinical roles that a normal and altered p53 protein play in cancer remain areas of intense investigation and debate, a number of studies have shown that alterations in p53 are either associated or not with patient outcomes, such as response to therapy or survival [2].

In this context, we present two case reports of very low-risk and low-risk MDS, according to the Revised International Prognostic Scoring System (IPSS-R), with two *TP53* gene mutations described for the first time in patients with MDS and we describe the outcomes of these patients.

Both patients are enrolled in a cross-sectional analytical study with adult patients of both genders diagnosed with low-risk MDS, receiving out-patient treatment at Walter Cantídio University Hospital. The differential diagnosis was performed in all patients. Peripheral blood samples were collected with ethylenediaminetetraacetic acid (EDTA) for laboratory analysis.

Initially, p53 protein expression of 38 patients with low-risk MDS was determined by immunohistochemistry [3, 4]. At a second stage, 37 patients were evaluated for mutations in the *TP53* gene by Sanger sequencing. Among these, only two patients had mutations. The study was approved by Walter Cantídio University Hospital Ethics Committee. Informed consent was obtained from all patients. Other causes of clonal or non-clonal cytopenias were excluded. Common etiologies of cytopenias or morphological abnormalities that may mimic MDS, such as aplastic anemia or paroxysmal nocturnal hemoglobinuria were investigated.

Molecular analysis of the *TP53* gene was performed at the Laboratory of Molecular Biology of the Bone Marrow Transplant Center (CEMO), Cancer Institute (INCA) in Rio de Janeiro, by direct sequencing (Sanger sequencing). Four pairs of primers were used for complete coverage of exons: 3–9; primer exon 3–4, primer exon 5–6, primer exon 7, and primer exon 8–9.

Exons 3–9 of the gene were amplified by polymerase chain reaction (PCR) in deoxyribonucleic acid (DNA) extracted from leukocytes. The PCR primers and conditions for amplification of genomic DNA followed those established by the International Agency for Research on Cancer (p53.iarc.fr/ProtocolsAndTools.aspx). All PCR products were confirmed by 1.5% agarose gel, purified using Wizard SV Gel kits and PCR Clean-Up (both by Promega) and sequenced in a 16-capillary automated sequencer (ABI PRISM® 3100 Genetic Analyzer, Applied Biosystems). The sequence data files were analyzed using Mutation Surveyor (SoftGenetics) software. All variants found were compared with the databases: COSMIC, dbSNP, and 1000 genomes UniProtKB.

## Case presentation

### Case report 1

**Fig. 1:**
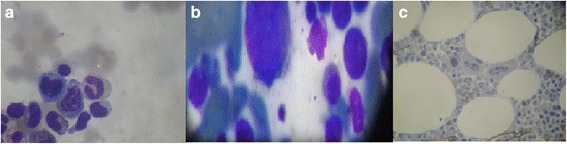
Myelogram of patient 1. **a** Dyserythropoiesis; **b** dysmegakaryopoiesis; **c** reticulin stain

In 2011, a 77-year-old Brazilian woman was admitted to our service due to refractory anemia. She was treated with vitamin B12, folic acid, and iron, but she showed no improvement. A bone marrow aspirate (5 January 2011) showed dyserythropoiesis greater than 20%, dysgranulopoiesis greater than 20%, and 4% of blasts; her iron level was normal. A second bone marrow aspirate (8 June 2011) showed hypocellularity with dyserythropoiesis and dysgranulopoiesis (less than 10%), 1% of blasts, and absent iron. A third myelogram (18 August 2013) showed mild dysmyelopoiesis, hypocellularity with 7% dysmegakaryopoiesis, and moderate dysgranulopoiesis (12%); there was also a reduced deposit of iron and 0.5% of blasts (Fig. 1). The first bone marrow biopsy (8 June 2011) showed hypocellularity, 20% of dyserythropoiesis, abnormal localization of immature myeloid precursors (ALIPs), and dysplastic megakaryocytes. The second bone marrow biopsy (12 August 2013) showed hypocellular bone marrow with dysmegakaryopoiesis and dyserythropoiesis associated with myelofibrosis degree II. The immunohistochemical studies of p53 expression and anti-CD34^+^ were negative. Serology for hepatitis B, hepatitis C, human immunodeficiency virus (HIV), and human T-cell lymphotropic virus (HTLV) were negative. Her thyroid hormones, ferritin, and dehydrogenase lactate levels were normal. A differential diagnosis for aplastic anemia was performed. Cytogenetic analysis showed a normal female karyotype. She was diagnosed as having multiple lineage dysplasia MDS, according to World Health Organization (WHO) 2016 and classified as very low risk based on IPSS-R (score-1). She currently shows no clinical or laboratory manifestations of the disease (Table 1).

### Case report 2

**Fig. 2:**
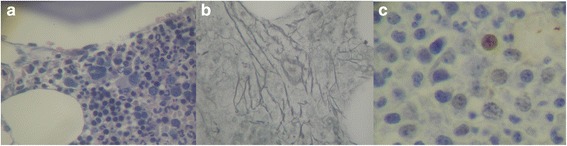
**a** Myelogram of patient 2. **a** Hypercellularity; **b** mild focal thickening with reticulin staining; **c** p53 expression by immunohistochemistry at 2%

In 2011, an 80-year-old Brazilian man, presented to our hospital with anemia, thrombocytopenia, and monocytosis (>1000/mm^3^). He had dizziness and normal blood pressure levels. A myelogram showed dyserythropoiesis, dysgranulopoiesis, and dysmegakaryopoiesis; blasts were approximately 2.2% (Fig. 2). Cytogenetic analysis showed a normal karyotype. A bone marrow biopsy showed hypercellularity with significant expansion of granulocytic and megakaryocytic series. Mild focal reticulin thickening was observed. He had not received any blood transfusions and had no infection reports. Routine examinations, including renal function and electrolytes, were within the normal ranges. An immunohistochemistry analysis showed normal expression of precursor cells; there was a moderate increase in vasculature and nuclear positive reaction to p53 (2%). The diagnosis was MDS with multiple lineage dysplasia. He was classified as low-risk MDS, according to IPSS-R (score-2.2 (Table 1)).

Currently, he is 85 and has difficulty walking, but with a good overall health status, despite his limitations. In both cases, patients with these mutations have had a good evolution despite a positive p53 expression.

## Discussion

P53 protein is a key regulator of stem cell homeostasis that impacts on the array of cellular functions including genomic surveillance, cell cycle regulation, and apoptotic and inflammatory response [5].

Inactivation of p53 has been reported in hematologic malignancies in association with disease progression. In adult patients with *de novo* MDS or acute myeloid leukemia (AML), somatically acquired *TP53* mutations are observed in approximately 10% of cases, and are often associated with loss of the short arm of chromosome 17, a complex karyotype, resistance to chemotherapy, and a short survival [2, 6].

**Fig. 3:**
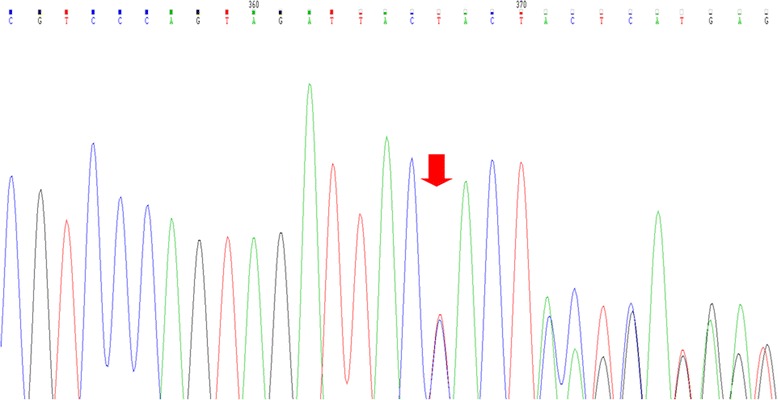
Electropherogram of c.783-1_784delGTG mutation in exon 8 of case report 2. The *red* arrow is pointing the TP53 region where it has been observed the change on reading frame due to c.783-1_784delGTG mutation detected by Sanger sequencing

In our case reports, the patients were low risk and very low risk and both had a normal karyotype. Case 1 exhibited c.394A > T (p.K132*) nonsense mutation, which generates a non-functional protein [7]. Case 2 had a novel *TP53* c.783-1_784delGTG mutation (Fig. 3).

P53 normally interacts with a variety of proteins involved in transcriptional regulation, DNA repair, cell cycle progression, apoptosis, and proteasome-mediated protein degradation. Although the biological and clinical roles that normal and altered p53 play in cancer remain areas of intense investigation and debate, a number of studies have shown that alterations in p53 are either associated or not with patient outcomes, such as response to therapy or survival [8, 9].

In both cases, mutations were found in low-risk MDS. Considering the impact of *TP53* gene on hematologic malignancies, these two mutations may or may not contribute to the knowledge of the role of *TP53* in disease pathogenesis, tumor progression, and to the identification of new therapeutic targets.

## Conclusions

The present study discloses a surprising outcome to the scientific community. The *TP53* c.783-1_784delGTG mutation has been identified for the first time. Regarding the other mutation, p.K132*, it has been considered in other types of tumors as a poor prognostic factor; in this specific case, it does not seem to be associated with complications regarding the patient’s clinical condition. This is a relevant result and more studies are needed to evaluate the role of these mutations in MDS.
